# Development of a Conceptual Framework for Art Making Intervention in Chronic Pain Management: A Narrative Review

**DOI:** 10.1093/geroni/igaf122.1483

**Published:** 2025-12-31

**Authors:** Kyung Soo Kim, Clara Baldus, Harleah Buck

**Affiliations:** University of Iowa, Iowa City, Iowa, United States; University of Iowa, Iowa City, Iowa, United States; University of Iowa, Iowa City, Iowa, United States

## Abstract

Chronic pain is a significant public health concern requiring holistic, multidimensional interventions. Art making has emerged as a promising approach to pain management, yet its theoretical foundations remain underdeveloped. Establishing a clear framework is essential for advancing research and ensuring scientific rigor. This narrative review synthesizes existing literature to explore how art making influences chronic pain and proposes a framework for its potential mechanisms. We conducted a narrative review with thematic analysis using a predetermined codebook. A systematic search of Medline, CINAHL Plus with Full Text, and Art Full Text identified articles published from January 2010 to March 2024. Studies were included if they examined art making interventions aimed at improving health outcomes such as pain or mental health and discussed their theoretical foundations. Exclusion criteria included a focus on art appreciation, non-English language, or lack of full-text availability. From 21 identified articles, 19 addressed theoretical foundations of art making interventions. Most studies (n = 12) relied on a single predetermined concept, such as creation of art, creativity, self-expression, or distraction, to explain their intervention’s foundation. This review suggests that art making can improve the multidimensional pain experience: ‘distraction’ may alleviate the physical aspect of pain, while ‘self-expression’ supports psychological well-being. However, the role of ‘creativity,’ a key factor in subjective well-being and neuroplasticity, remains underexplored. Further research is needed to integrate creativity into a more comprehensive theoretical framework for chronic pain management.

